# Caesarean Section Rates Among Syrian Refugees in Lebanon

**DOI:** 10.7759/cureus.81547

**Published:** 2025-03-31

**Authors:** Mahmoud El hussein, Mariana EL Helou, Charif Khaled, Jad Kassem, Sara Rachid, Jean Claude Estephan, Cima Hamieh

**Affiliations:** 1 Emergency Medicine, Lariboisière Hospital, Paris, FRA; 2 Emergency Medicine, Lebanese American University (LAU) Medical Center, Beirut, LBN; 3 General Surgery, Geitawi Hospital, Beirut, LBN; 4 Cardiology, American University of Beirut, Beirut, LBN; 5 Family Medicine, Rafik Hariri University Hospital, Beirut, LBN; 6 Anesthesia, Notre Dame de Secours, Beirut, LBN; 7 Family Medicine, Lebanese American University (LAU) Medical Center-Rizk Hospital, Beirut, LBN; 8 Geriatrics, Hôpital de Montfermeil, Montfermeil, FRA

**Keywords:** c-section, middle-east, refugee care, statistial analysis, women and health

## Abstract

Objectives: Deliveries can be complicated by many factors, especially if women are not well followed up. Syrian refugees, for the last decade, have had to go through unbearable circumstances, which have affected their health. This is evident in the pregnancy data when we look at the delivery methods and outcomes. The caesarian section (C-section) rate among Syrians before the civil war was as per the WHO recommendation; this rate, however, changed enormously for the Syrian refugees in Lebanon. This study aims to determine the C-section rate among Syrian refugees in Lebanon, identify contributing factors, and compare it to pre-war Syrian rates.

Study design: This retrospective study featuring a sample size of 2183 women was conducted in a government hospital, where refugees are hospitalized under the coverage of the United Nations High Commissioner for Refugees ( UNHCR). The studied variables include the mode of delivery, maternal age, parity, day of the week, maternal hospital stay, gestational age, sex of the baby, and weight of the baby.

Results: The C-section rate increased dramatically after the displacement of the refugees to Lebanon. This increase, however, is significantly lower than the rate among the Lebanese population. The main variables associated with an elevated C-section rate were maternal age (p-value <0.0001), day of the week (p-value <0.0001), gestational age (p-value <0.0001), and male babies (p-value = 0.024). Furthermore, C-section was associated with a longer hospital stay.

Conclusion: The C-section rate among Syrian refugees in Lebanon increased dramatically compared to the C-section rate in the Syrian population before the war. This increase is double the optimal rate recommended by the WHO.

## Introduction

The cesarean section (C-section) rate has been rising worldwide for more than two decades [1]. Lebanon, a small Mediterranean country, has one of the highest C-section rates (35% to 40%) compared to the Middle East and North Africa (29.6%)and even worldwide (21.1%) [2,3]. Syria, a neighboring country, had a C-section rate of 15 % before 2011 [4]. The Syrian civil war started in 2011, and refugees flooded to the surrounding countries to escape the crisis and seek asylum [5,6]. The United Nations High Commissioner for Refugees (UNHCR) reported around one million registered Syrian refugees in Lebanon in 2018, equivalent to about 17% of the Lebanese population [6]. Women represent 52.5% of this number. Unfortunately, around 76% of the refugees in Lebanon live below the poverty line and lack adequate medical care [7]. Women found it hard to reach care centers and faced problems while dealing with the Lebanese healthcare system [8,9,10]. In particular, Syrian pregnant women lack the needed follow-up, and are at risk of poorer maternal outcomes and increased maternal and fetal complications [11,12].

Several studies were conducted among Syrian refugees in Lebanon to ascertain their situation and needs. Few were aimed at the maternal health care [8,11-14]. Data showed that only 54% of pregnant women had an initial maternal check-up in the first trimester, with an average of 4.8 antenatal care (ANC) visits [12]. Many of them did not receive adequate maternal follow-up. The most common locations where Syrian pregnant women consult were a primary health care center (54.9%) or a private clinic (42.6%) [12]. The cost was the primary reason why pregnant women did not receive ANC [12]. Furthermore, Syrians were met with a predicament as they were almost always received by male doctors [4]. Finally, even when they surpassed all these difficulties, they were met with extreme prejudice from healthcare employees [10]. All of this led to poorer ANC visits compared to other countries [11].

Very few studies have been conducted on studying the C-section rate among Syrian Refugees in Lebanon [8,12]. The few studies that do exist estimate the C-section rate in Lebanon to be around 40% [8,12]; this rate is higher than the WHO recommendation [10]. Our study was conducted to assess the maternal characteristics of pregnant Syrian refugees. Its objective is to assess the C-section rate among Syrian refugees in Lebanon, compare it to pre-war Syrian rates, identify factors influencing this rate, and finally, to describe the neonatal characteristics of the delivered babies.

## Materials and methods

This is a retrospective cohort study. It was approved by the hospital management committee. An institutional review board (IRB) approval was granted by the IRB committee of the Lebanese University. 

Data about pregnant Syrian refugees that presented to the maternity ward of Bouar Governmental Hospital (Bouar, LBN), between January 1, 2014 and December 31, 2018 were collected. The sample size was 2183 pregnant women. All patient files were retrieved from the archives. The record of each patient was reviewed and analyzed.

The variables studied were maternal age, parity, delivery mode, day of the week, maternal hospital stay, gestational age, sex of the baby, and weight of the baby. This data was easy to find in patients’ charts. The only limitation was the difficulty in accessing the information in some files where data was missing; these files were omitted.

Participants were selected through convenience sampling due to the limited access to Syrian refugees in Lebanon. Inclusion criteria were Syrian refugee women who had given birth in Lebanon within the past year. Exclusion criteria were women with incomplete medical records or pre-existing medical conditions that could affect the mode of delivery, or complications during pregnancy/delivery such as stillbirth. 

Quantitative variables were described by their means, medians, and standard deviations (SD). The qualitative variables were described by their respective percentages. Advanced statistical analysis was conducted using SPSS Statistics version 25 (IBM Corp., Armonk, NY, USA). The chi-square test and t-test were performed to determine if any significant correlation was present between the studied variables. The dependent variable is the delivery method, namely the C-section and natural vaginal delivery (NVD). Relevant variations were considered as independent variables. Logistic regression was done to determine if the independent variables had any impact on the mode of delivery. The results were divided into three sections: maternal characteristics of UNHCR registered patients, pregnancy outcomes of UNHCR patients, and neonatal characteristics for each delivery mode. A p-value < 0.05 was considered significant.

## Results

Maternal characteristics of UNHCR registered patients

The average maternal age at childbirth was 25.56 + 6.16 years. Data showed that 1758 patients (80.53%) were between 18 and 35 years, while 162 (7.42%) women were underaged, reaching the very young age of 11 years. The group of over 36 years consisted of 156 patients (7.15%). The average age for C-section patients was 26.4 +/- 6.21 years, while that of NVD patients was 25.18 +/- 6.11 years.

Also, the C-section rate seemed to increase with age. Older age groups showed a higher C-section rate, reaching 37.18% for women ≥36 years old. On the other hand, women ≤17 years old had a much lower C-section rate of 22.22% (p-value = 0.0918). Note, this result shows that the rate tends to increase as women get older, but the p- p-value indicates that the difference between the two age groups is not statistically significant. 

The average parity was 2.62 +/- 1.73. The minimum was one delivery per woman, and the maximum was 13 deliveries per woman. In total, 1650 women, which is around three-quarters (75.68%) of the women, were ≤ P3, and 11.91% of them were ≥P5. Further analysis showed that C-section patients had a lower parity average than NVD patients (p-value = 0.7).

Pregnancy outcomes of UNHCR patients

The C-section rate varied throughout the years, with an average of 31.37%. All indications of C-sections are included in our results, i.e., elective and during labor. They reached a minimum of 26.2% in 2016 and a maximum of 38.8% in 2017. The C-section rates significantly varied within the same week (p-value <0.0001). They hit the highest rates on weekdays (34.15%) and the lowest on weekends (16.89%). 

The average hospital stay for women was 1.55 +/- 0.58 days (Table 1). The minimum was one day, and the maximum was five days. Most patients stayed between one and two days. Data analysis showed that the hospital stay difference between the two patient groups (NVD vs. C-section) was statistically significant (Table 1). Following a C-section delivery, 88.48% of patients stayed for two days or more, and a minority of 11.52% stayed for one day. On the other hand, around two-thirds (64.25%) of NVD patients stayed for one day. 

**Table 1:** Hospital stay characteristics for each delivery mode NVD: Natural vaginal delivery, CS: Caesarean section

Hospital stay	NVD	CS	P-value
Count	1503	680	<0.001
Average	1.37	2.01	
SD+	0.51	0.49	

Neonatal characteristics of babies of UNHCR patients

The average gestational age was 38.82 +/- 1.16 weeks (Table 2). The minimum was 28 weeks, and the maximum was 43 weeks. Only 36 babies (1.65%) were born preterm.

**Table 2:** Gestational age characteristics for each delivery mode NVD: Natural vaginal delivery, CS: Caesarean section

Gestational age	NVD	CS	t-score	p-value
Count	1503	680	-4.34	<0.001
Average	38.56	38.82		
SD +	1.11	1.18		

The average gestational age upon delivery for NVD patients was lower than that of C-section patients, with a p-value <0.0001. The sex of the newborn was studied to see whether it had any impact on the delivery mode. Both sexes were almost equal, with a slight predilection for male babies (51.31%). Interestingly, male babies required C-section delivery (33.75%), significantly more than female babies (28.41%) (p-value = 0.0071).

The average baby weight was 3091 g+ 441. The minimum was 1500 g, and the maximum was 5000 g. Around 151 babies (7%) were born with low birth weight (LBW). Furthermore, results showed that women carrying LBW babies required C-section more often (33.77%) than women carrying babies ≥ 2500 g, but these differences were statistically insignificant (p-value = 0.47).

Further analysis of the sex of the babies delivered showed that male babies had a higher birth weight average than female babies. After performing logarithmic regression, we concluded that the major risk factors and predictors for C-section were the day of the week that the woman presented on, the age of the woman, the gestational age, and the sex of the baby (Table 3).

**Table 3:** Factors associated with CS CS: Caesarean section, OR: Odds ratio, CI: Confidence interval

Variables	OR	95% CI	Z statistic	p-value
Weekday	2.55	1.92-3.4	6.425	<0.0001
Term	3.68	1.3-10.44	2.446	0.0145
Sex	1.28	1.07-1.54	2.69	0.0071
Age	1.34	0.95-1.87	1.683	0.0924
Parity	1.1	0.89-1.36	0.864	0.3878
Weight	1.14	0.80-1.61	0.722	0.4706

## Discussion

Syrian refugees live below the poverty line, and many of them have no access to basic life needs, and in particular, health needs. A systematic review conducted in 2019 on displaced Syrian refugees reported that major gaps were found in general healthcare and women’s healthcare services [15]. This tough living situation led them to seek cheaper healthcare facilities [7]. For delivery, the most common location was in a hospital, and the choice of hospital depended mainly on the cost [12]. At the Bouar Government Hospital, the patients were able to be hospitalized under the coverage of the UNHCR while nearly all the other hospitals in the area were private. Hospitalization in the private sector costs two to three times more than that of the public sector. 

Despite the increased C-section rate in Lebanon (40%), the C-section rate among the UNHCR patients in our study was significantly lower (31%) [11]. In addition, financial difficulties had an impact on the ANC. It has been shown that in Lebanon, there is a group of Syrian women not receiving ANC, with expenses being the primary burden (75.9%) [11]. The C-section rate among Syrian refugees remained higher than the rate recommended by the WHO and higher than the C-section rate in Syria before the civil war.

The C-section rate varied throughout the years in this study, but the rate was always higher than 15%. The C-section, when indicated, can be lifesaving for both mother and newborns. However, over-indication can lead to increased fetal and maternal complications [2]. The indications were similar in most studies. The most common indication was a previous C-section. Other indications included baby malpresentation (breech, transverse) and primary C-section due to lack of follow-up.

The average hospital stay was higher for C-section patients, as the women required closer postoperative monitoring. A woman is usually hospitalized for three days following a C-section, which was not the case here. Most patients were discharged on day one post-C-section. This short hospital stay is mainly due to financial issues. Furthermore, this could lead to increased morbidity as the mothers are not adequately followed up.

All reviewed studies, including ours, report that increased age was a risk factor for C-section. Our data shows that the median age for Syrian refugees undergoing deliveries (25.56 years) was lower than the number found in the literature for Lebanese women (28.5 years) [12]. One of the reasons that contributes to refugees having a younger maternal age compared to natives is mainly a monetary reason. Due to the financial burden, displacement, and lack of proper education, Syrian refugees were encouraged to get married at a very young age [13-14]. Early marriage was the cause of the increased rate of underage mothers among refugees. 

No straight correlation was found between the parity and the delivery mode. Nevertheless, the parity contributed to repeating the primary delivery mode, thus increasing its rate, as P2 women are usually delivered the same way as their first delivery. Many studies interpret the cause of increase in parities was that Syrian refugee families preferred that their daughters marry at an early age as a way to protect them [15].

The fetal gestational age affected the NVD rate rather than the C-section rate. Syrian refugees had a low preterm percentage (1.65%), although there was no proper follow-up of these pregnant women during pregnancy. Our study also shows that male babies had a significantly higher birth weight. However, the sex is not a controlled variable, but proper ANC could lead to better preparation for any delivery.

This study shows normal birth weight for refugee babies in 93% of deliveries. The average weight was 3091 g. Even though the low birth weight category was small, Syrians had lower extremes of birthweight. A recent study by Benage et al. showed that only 41% of Syrian women in Lebanon have complete nutritional support [13].

Limitations

Our study was able to reflect the numbers in C-section delivery among Syrian refugees; however, it can not account for the missing or incomplete data and illegal, unrecorded procedures. It also does not account for the mother's choice in opting for a C-section, as no contact was made with the mothers. Additional limitations of the study is one, the potential for response bias, as participants may have provided socially desirable responses regarding their experiences with C-sections as Syrian refugees in Lebanon; and two, the inability to establish causal relationships between C-section rates and the identified risk factors due to the observational and retrospective nature of the study.

## Conclusions

This study concluded that many factors affect the delivery method among Syrian refugees; maternal age, day of the week, gestational age, and sex of the baby were the most significant. Nevertheless, the C-section rate among Syrian refugees in Lebanon was lower than that of the Lebanese population. Despite that, the result remains double the recommended rate. The role of the WHO and Lebanese health authorities is to spread awareness among the refugees about delivery options, the risks, and the outcomes of a C-section. It is also essential to follow up on these subjects and look at the rates and type of complications they might have endured post-C-section. 
